# A commentary on ‘Intraoperative endomanometric laparoscopic Nissen fundoplication improves postoperative outcomes in large sliding hiatus hernias with severe gastroesophageal reflux disease: a retrospective cohort study’

**DOI:** 10.1097/JS9.0000000000001177

**Published:** 2024-02-08

**Authors:** Tingfen Han, Shuai Liu, Xiasang Qiu

**Affiliations:** a Department of Traditional Chinese Medicine; b Department of Gastrointestinal Surgery, Taizhou Hospital of Zhejiang Province Affiliated to Wenzhou Medical University, Taizhou, People’s Republic of China

*Dear Editor*,

Gastroesophageal reflux disease (GERD) is a disease that occurs in the esophagus or beyond due to the reflux of gastric contents^1^. Esophageal hiatal hernia, most commonly type I sliding hiatal hernia (HH), is one of the leading causes of GERD^2^. Laparoscopic Nissen fundoplication (LNF) is currently recognized as the preferred surgery for the treatment of GERD^3^. Habeeb *et al*.^4^ conducted a retrospective multicenter cohort study designed to assess the impact of intraoperative endoscopy and intraoperative manometry in improving prognosis after LNF.

The authors clearly defined the concepts of severe GERD (DeMeester score >100) and large sliding hiatal hernias (HHs) (>5 cm). The design of this study, which focused on the intraoperative use of high-resolution manometry (HRM) and endoscopy to improve surgical outcomes and patient satisfaction, is commendable as it attempts a less-explored area in GERD.

However, there are still some limitations:

First, pneumoperitoneum is a key factor in laparoscopic surgery, so it is necessary to record the pneumoperitoneal pressure for each patient^5^. Consideration also needs to be given to the effect of pneumoperitoneal pressure on the actual lower esophageal sphincter (LES) pressure values measured during the procedure and how to eliminate the effect of this pressure.

Second, there is no consensus on the target pressure value for intraoperative LES. The present study is commendable for setting intraoperative targeted LES pressure values (20–40 mmHg). The authors should add the basis for setting the target values, such as clinical experience or evidence-based medicine, to increase the rigor of the article. In addition, the threshold range of 20–40 mmHg fluctuates greatly, and further subgroup analysis can be conducted.

Third, LNF as a function-improving surgery, patient health/quality of life, and satisfaction are important outcome indicators to assess efficacy. However, due to the differences in physical tolerance and educational background of different patients, their subjective feelings and verbal descriptions are sometimes inconsistent with the objective results, which can easily lead to biased results for symptoms such as dysphagia and reflux. Therefore, it is necessary to introduce objective indicators. The annual postoperative endoscopy in this study was an objective indicator to aid in validating subjective results. Suggest researchers further record endoscopic manifestations, such as inflammation, erosion, edema, hyperplasia, or other conditions. It can more accurately reflect the postoperative efficacy. If possible, the addition of 24-h pH monitoring and measurement of pressure in the high-pressure zone of the distal esophagus would have resulted in a more comprehensive assessment.

Fourth, all patients in this study had the same anesthesia regimen, and the surgeries were performed by experienced surgeons. However, since anesthesiologists and surgeons are not always the same, there are differences in surgical outcomes due to operational subjectivity. Therefore, the standardization of LNF should also be considered. Meanwhile, the experience and learning curve of the surgeons can be evaluated and correlated with the surgical outcomes.

I highly appreciate the author’s innovative approach to incorporating endoscopy and HRM into the study of GERD. Considering the above suggestions can make the details of the article more comprehensive.

## Ethical approval

This manuscript is a comment. Don’t need ethical approval.

## Consent

This manuscript is a comment. Don’t need consent.

## Sources of funding

None.

## Author contribution

T.H.: investigation and writing – original draft; S.L.: conceptualization; X.Q.: supervision and writing – review and editing.

## Conflicts of interest disclosure

There are no conflicts of interest in our commentary.

## Research registration unique identifying number (UIN)

None.

## Guarantor

Xiasang Qiu.

## Data availability statement

None.

## Provenance and peer review

None.
